# Can men’s violence reporting behavior be improved? Evidence from a couples-based cluster randomized controlled trial in Tanzania

**DOI:** 10.3389/fpubh.2025.1460333

**Published:** 2025-11-12

**Authors:** Nafisa Halim, Neeta Ghanekar, Ester Steven Mzilangwe, Naomi Reich, Lilian Badi, Nandini Agarwal, Lisa Messersmith

**Affiliations:** 1 Department of Global Health, School of Public Health, Boston University, Boston, MA, United States; 2 Muhimbili University of Health and Allied Sciences, Dar es Salaam, Tanzania; 3 World Education Inc./Bantwana Initiative, Boston, MA, United States; 4 World Education Inc./Bantwana Initiative Tanzania, Arusha, Tanzania; 5 Department of Psychiatry, Boston Medical Center, Boston, MA, United States

**Keywords:** intimate partner violence, survey, self-report, validity, resource-limited settings

## Abstract

**Purpose:**

Intimate partner violence (IPV) is challenging to measure due to underreporting by both men and women. The reasons why men fail to disclose their IPV perpetration in societies where such behavior is more socially accepted remain an open question. Men’s lack of awareness of how their behaviors can harm women is a contributing factor. We evaluated the impact of an intervention designed to increase men’s understanding of IPV on their ability to report it accurately, as indicated by men reporting IPV consistently with their female partners (whose reports are valid indicators of IPV).

**Methods:**

We analyzed data from a cluster-randomized controlled trial conducted in Tanzania. A total of 450 couples from nine villages were randomly assigned to one of three arms, with each arm comprising 150 couples. In Intervention Arm 1, men participated in peer groups that explored gender relations and IPV. In Intervention Arm 2, men participated in peer groups, and communities additionally engaged in dialogues on similar topics. The Control Arm had no such activities. IPV data were collected from both partners, following ethical guidelines to ensure women’s safety. Our primary outcome includes couples reporting concordantly whether IPV occurred (couples’ concordant reporting).

**Results:**

From baseline to endline, concordant reporting among couples increased for physical (31%, *p* = 0.002), sexual (24%, *p* = 0.01), and economic (22%, *p* = 0.05) IPV in Intervention Arm 1, accompanied by fewer men disagreeing with their female partners’ IPV reports. Similarly, in Intervention Arm 2, concordant reporting increased for physical (24%, *p* = 0.02) and sexual (22%, *p* = 0.04) IPV. There was no improvement in the concordant reporting of emotional IPV across the arms. Compared to couples in the Control Arm, those in Intervention Arm 1 had higher odds of reporting concordantly on physical (OR: 1.85, 95% CI: 0.97, 3.51, *p* = 0.09) and economic (OR: 1.83, 95% CI: 0.96, 3.46, p = 0.09) IPV at endline compared to baseline.

**Conclusion:**

In communities that do not link IPV to fault, men may not report IPV because they do not recognize their behaviors as abuse. Including a preamble that defines IPV in a survey questionnaire can improve men’s reporting of IPV.

## Introduction

Women’s self-reports are commonly used to identify millions of women worldwide who experience intimate partner violence (IPV), which includes physical aggression, sexual coercion, psychological abuse, or economic deprivation carried out or attempted by a current or former romantic partner to control them (1, 2). Recognizing these women is crucial, as they may need safety planning, mental health treatment, and legal support to address the wide-ranging impacts of IPV, such as depression, anxiety, and substance use (1, 2). However, relying on self-reports carries the risk of misclassifying victimization as non-existent since not all women disclose IPV due to fears of retaliation, such as abandonment or losing custody of children (3, 4). Therefore, addressing underreporting is vital for improving the accuracy of self-reports in IPV prevention and response. Since self-reports tend to be more reliable than police or hospital records, they are likely to remain the primary source of IPV data for the foreseeable future (5, 6).

Ethically collected self-reported data from female (f) on IPV victimization and from male (m) partners on perpetration, respectively, can help address unreported IPV (7, 8). A woman’s report that she has experienced IPV is a well-established indicator of her IPV victimization (Table 1, Cells 1 and 2) in IPV research (1, 9–11), regardless of whether her male partner agrees (Table 1, Cell 1, *yes–yes* concordance from now on) or disagrees (Cell 2, *yes–no* discordance from now on) with her report. Similarly, a man’s report of his IPV perpetration is a well-established indicator of his IPV perpetration and his female partner’s IPV victimization (Cells 1 and 3) (12), regardless of whether his female partner agrees (Cell 1, *yes–yes* concordance) or disagrees (Cell 3, *no–yes* discordance from now on) with his report. Therefore, leveraging discordance in IPV reporting within couples (8, 13) allows researchers to identify more women experiencing IPV by using reports from both partners rather than relying solely on women’s reports.

**Table 1:** Women’s and their male partners’ reporting of IPV.

IPV Reporting		Male partners’ reporting of IPV perpetration:	
		*Yes, I did*	*No, I did not*
Female partners’ reporting of IPV victimization:	*Yes, I did*	1	2
	*No, I did not*	3	4

While promising, the above approach relies on the ability to reduce discordance to a reasonable level (7, 14, 15). This is because high discordance may indicate measurement errors arising from current data collection methods (i.e., IPV questions can have different meanings for women and men, leading to inconsistent reports) (16, 17). Notably, discordance can reach as high as 50%, with women reporting up to 50% more IPV incidents than their male partners in several low-income countries (7, 15, 18, 19). In countries where IPV is widely accepted (20), high discordance in IPV reports within couples is surprising, as men are often willing to disclose their perpetration behaviors without fearing negative sanctions (7, 21). This raises concerns about the validity of assumptions and methods used in IPV data collection from men. For example, definitional differences between genders—such as when a woman considers a behavior (e.g., forced sex) as IPV, but her male partner does not—probably contribute to discordance (7, 17). Also, experiential differences—such as women recalling past abuse more accurately than men, since IPV is traumatic for women and routine for men—may also influence the results (13, 17).

Therefore, we ask in this brief report: *Can concordance in couples’ IPV reporting be improved by improving men’s understanding of IPV?* If concordance is indeed improved, suggesting dwindling discordance, *to what extent is this improvement due to a decrease in yes–no discordance, indicating an improvement in men’s reporting of their violent behavior*? To answer these questions, this study uses data from a cluster randomized controlled trial (RCT) conducted in 2015–2016 to test the preliminary efficacy of a gender transformative training intervention for men and community members in Tanzania. Informed by the Socio-Ecological Model and Social-Cognitive Theory, the intervention provided IPV education to men through 24 h of male peer-group workshops and two-day-long community dialogues. We hypothesized that by *participating in male peer-group workshops and community dialogues, men would gain a better understanding of IPV in its* var*ious forms, which would enable men to recognize their abusive behaviors and encourage them to report their perpetration behavior, leading to a decrease in yes–no discordance and an increase in couples’ concordant reporting*.

## Methods

We analyzed data from 450 couples who were involved in the trial mentioned above. The trial involved a pilot cluster RCT, where nine villages were randomly assigned to one of the three arms using a pseudo-random number generator. In the Control Arm, women participated in livelihood improvement groups, but their male partners received no direct intervention. In Intervention Arm 1, women participated in livelihood improvement groups, while their male partners received training in gender relations and IPV through male peer groups. All men in this arm received training for a total of 24 h. In Intervention Arm 2, women participated in livelihood improvement groups, their male partners participated in male peer groups, and community members participated in two-day-long gender dialogues. In each arm, the study recruited the first 150 couples that were screened as eligible. Women were considered eligible if they were at least 18 years old, living with a male partner, could provide consent to participate in the interviews, and gave permission to study personnel to contact their male partner about the trial. The male partner was also required to provide consent to participate in the interventions and interviews.

### Ethics

Ethical approvals were obtained prior to data collection. All participants completed the informed consent process prior to the interview. There was a protocol for flagging if women appeared upset by the interview questions, and a social welfare officer was on site during all the interviews to speak with women and manage referrals to social services, as needed.

### Data collection

The baseline interviews took place in July 2015, preceding the implementation of the intervention from August 2015 to March 2016. Endline interviews were completed in April and May 2016. Same-gender enumerators—trained over 2 days on ethics, sensitive research topics (e.g., IPV), informed consent, and survey research—interviewed women and men separately in a private location.

### Tools

We used the WHO Multi-Country Study on Women’s Health and Domestic Violence Instrument (22) to collect data on physical, sexual, emotional, and economic IPV perpetration and victimization at baseline and endline. We used the Childhood Trauma Questionnaire (23) to assess childhood trauma and the Gender-Equitable Men (GEM) Scale (24) to measure gender-equitable beliefs.

### Measures

#### Outcomes

We had two outcome variables: couples’ concordant reporting (primary) and changes in concordance (secondary). For measuring concordant reporting, we used men’s and women’s reports of IPV perpetration and victimization, respectively. We classified men as having perpetrated physical, sexual, emotional, and/or economic IPV if they reported committing *once*, *a few times*, or *many times* any one of the five acts of physical IPV (*slapped a partner or thrown something at her that could hurt her*; *pushed or shoved a partner*; *hit a partner with a fist or with something else that could hurt her*; *kicked, dragged, beaten, choked or burned a partner*; *threatened to use or actually used a gun, knife or other weapon against a partner*), the two acts of sexual IPV (*forced a partner to have sexual intercourse when she did not want to*; *forced a partner to do something sexual that she found degrading or humiliating*), the five acts of emotional IPV (*insulted a partner or deliberately made her feel bad about herself; belittled or humiliated a partner in front of other people; done things to scare or intimidate a partner on purpose; threatened to hurt a partner; hurt people she cares about as a way of hurting her, or damaged things that are important to her*), or the five acts of economic IPV (*prohibited a partner from getting a job, going to work*; *trading or earning money*; *taken a partner’s earnings against her will*; *thrown a partner out of her house*; *or kept money from a partner’s earnings for alcohol, tobacco or other things knowing that partner was finding it hard to afford household expenses*), included in the WHO survey. Similarly, we considered women to have been victimized by physical, sexual, emotional, and economic IPV if they reported experiencing *once*, *a few times*, or *many times* any one of the five acts of physical IPV, the two acts of sexual IPV, the five acts of emotional IPV, or the four acts of economic IPV, included in the WHO survey. We measured couple concordance in IPV reporting as a binary variable: Couples received 1 if both female and male partners reported with a *yes* or a *no* to the questions on IPV perpetration and victimization in the last 3 months; couples received 0 if a male partner reported *no* to a question on IPV perpetration but his female partner reported *yes*, or if a male partner reported *yes* to a question on IPV perpetration but his female partner reported *no* to IPV victimization.

Finally, for each type of IPV, we constructed a change variable: Couples received a value of 1 if they reported concordantly at endline and discordantly at baseline; otherwise, couples received a value of 0.

#### Covariates

We measured 52 covariates at the men (18), women (19), couples (4), and household (11) levels on a categorical or continuous scale. We constructed a childhood trauma scale with scores ranging between 52 and 13: where higher the score, the more traumatic the childhood is (Cronbach’s *α* = 0.75), and men’s and women’s GEM scores ranging between 68 and 17: where higher the score, the more inequitable the attitudes will be [Cronbach’s *α* = 0.78 (men) – 0.74 (women)].

### Statistical analysis

We calculated the percentage of couples who reported concordantly (*yes–yes*; *no–no*) on physical, sexual, emotional, and economic IPV at baseline versus endline in the Control Arm, Intervention Arm 1, and Intervention Arm 2. Next, we calculated the percent of change in concordant (*yes–yes*; *no–no*), *yes–no* discordant, and *no–yes* discordant reporting for each of 16 acts of violence across four IPV types, among couples in the Control Arm, Intervention Arm 1, and Intervention Arm 2 from baseline to endline, to determine the source of the increase in couples’ concordant reporting, when applicable. Finally, we conducted an intent-to-treat (ITT) analysis via multilevel logistic regression models using the equation to estimate intervention effects on couples’ concordant reporting: $Y_{\mathrm{ij}}^{*}$ = $\beta \prime Z_{i}+v_{j}+\varepsilon_{\mathrm{ij}}$ , where Y^*^ denotes the log-odds of a vector of *y* outcomes capturing concordant reporting of physical, sexual, emotional, and economic IPV by couple *i* in village *j*. *Y_ij_* is 1 if the log-odds of concordant IPV reporting $Y_{\mathrm{ij}}^{*}$ > 0, 0 elsewhere, and $\beta \prime Z_{i}=$ *β*_1_*T_j_* + *β*_2_*X_ij_*. *T* is an indicator of random assignment to the Control Arm, Intervention Arm 1, or Intervention Arm 2; *X* is a vector of the covariates, namely baseline concordance, men’s school attendance, women’s school attendance, and household asset index. These variables were among the list of variables found to be different by study arm (see Sample Characteristics section below). We carried forward baseline values for men’s and women’s reports of IPV for missing endline ones as per recommendations for ITT analysis.

## Results

### Sample characteristics

Men had an average age of 41 years; 92% completed at least secondary education; 80% watched television as a form of mass media exposure; 29% used alcohol or other substances; and 28% had multiple sexual partners. On average, women were 36 years old at the time of the survey and 21 years old at the time of their first marriage. Approximately 90% completed at least secondary education, 33% read a newspaper, 29% watched television, 95% reported having one sexual partner, 10% used alcohol or other substances, 68% had been tested for HIV at some point, and 4% tested HIV-positive. Household size averaged four children: two males and two females. Approximately 44% experienced food shortages for 3–12 months per year, and 70% struggled to afford treatment or medicine.

### Randomization balance

Among the study groups, men differed in age and school attendance; women differed in age, television viewership, and HIV-positive status; and households differed in the number of male and female children, as well as in wealth.

### Survey attrition

Men: The attrition rates were 24.7% in the Control group, 13.3% in Intervention 1, and 18.7% in Intervention 2, averaging 18.9%. Women: The attrition rate ranged from 10.7% (Intervention 1) to 20.0% (Intervention 2), with an average of 16.2%. Overall, the randomization balance remained intact, as the men (*n* = 365) and women (*n* = 377) who remained in the study were comparable across all study arms.

### Descriptive findings

Table 2 shows the rate of couples’ concordant reporting at baseline and endline, broken down by study arm.

**Table 2:** Baseline characteristics in women, men and households across study arms, *n* = 450 couples, Tanzania, 2015–2016.

Variable	All		Control (*n* = 150)		Intervention 1 (*n* = 150)		Intervention 2 (*n* = 150)		*p*
	Mean/%	SD	Mean/%	SD	Mean/%	SD	Mean/%	SD	
Women’s characteristics:									
Current age (in years)	35.98	10.60	34.53	9.73	38.55	10.66	34.85	10.96	**
Age of first marriage (in years)	21.24	3.45	21.15	3.29	21.42	3.86	21.15	3.18	
% Woman was previously married (Ref: *no*)	19.33	39.54	30.67	46.27	14.67	35.50	12.67	33.37	***
% Husband has >1 wife (Ref: *Husband has 1 wife*)	10.67	30.90	12.00	32.61	12.67	33.37	7.33	26.16	
% Respondent is 1st/only wife (Ref: 2nd/3rd wife)	82.89	37.70	83.33	37.39	83.33	37.39	82.00	38.55	
% Marriage involved dowry/bride price (Ref: *no*)	55.78	49.72	55.33	49.88	60.00	49.15	52.00	50.13	
% Ever attended school (Ref: *no*)	90.89	28.81	92.00	27.22	88.67	31.81	92.00	27.22	
% Highest level of schooling (Ref: *Primary or none*)									
Secondary	76.67	42.34	74.00	44.01	78.67	41.10	77.33	42.01	
Higher	13.11	33.79	15.33	36.15	9.33	29.19	14.67	35.50	
% Reads a newspaper or magazine (Ref*: never*)	33.56	47.27	41.33	49.41	29.33	45.68	30.00	45.98	
% Listens to the radio (Ref: *never*)	62.89	48.36	60.00	49.15	63.33	48.35	65.33	47.75	
%Watches television (Ref: *never*)	29.33	45.58	40.67	49.29	18.00	38.55	29.33	45.68	**
% Heard of, seen or participated in campaigns or activities about IPV prevention in community or workplace (Ref: *no*)	56.00	49.69	65.33	47.75	44.67	49.88	58.00	49.52	*
% Number of sexual partners in last year ≤1 (Ref: ≥*2*)	94.67	22.49	92.00	27.22	96.67	18.01	95.33	21.16	
% Alcohol non-use (Ref: *daily, weekly* or *monthly use*)	89.56	30.62	85.33	35.50	90.00	30.10	93.33	25.03	
% Tested for HIV (Ref: *have not been tested for HIV/unknown or missing/refused to answer*)	67.78	46.78	70.00	45.98	70.00	45.98	63.33	48.35	
% HIV Positive (Ref: *HIV Negative/HIV status unknown/ refused to disclose status*)	4.44	20.63	9.33	29.19	3.33	18.01	0.67	8.16	**
Attitudes on gender norms and relations (GEM scores)	47.03	8.18	46.92	8.40	46.51	7.73	47.65	8.39	
Men’s characteristics:									
Current age (in years)	40.81	11.72	39.65	11.64	39.91	11.22	40.95	10.95	*
Age of first marriage (in years)	25.51	5.18	25.25	5.14	26.43	5.19	26.84	5.28	*
% Ever attended school (Ref: *no*)	92.00	27.16	90.67	29.19	98.00	14.05	98.36	0.13	**
% Highest level of schooling (Ref: *Primary or none*)									
Secondary	73.11	44.39	70.67	45.68	70.00	45.98	78.67	41.10	
Higher	18.67	39.01	20.67	40.63	17.33	37.98	18.00	38.55	
%Earned money in last 12 months (Ref: *no*)	97.11	16.77	96.67	18.01	97.33	16.16	97.33	16.16	
% Reads a newspaper or magazine (Ref*: never*)	0.62	0.49	0.67	0.47	0.52	0.50	0.67	0.47	**
% Listens to the radio (Ref: *never*)	88.67	31.74	89.33	30.97	84.00	36.78	92.67	26.16	
% Watches television (Ref: *never*)	80.89	39.36	81.33	39.09	77.33	42.01	84.00	36.78	
Childhood trauma	19.01	3.54	18.72	3.43	18.92	3.68	19.39	3.51	
% Condom non-use (Ref: condom use)	71.78	45.06	71.33	45.00	75.33	43.25	68.67	46.54	
% Multiple sexual partners (Ref: one sexual partner)	28.22	45.06	32.67	47.00	24.67	43.25	27.33	44.72	
% Alcohol or drug use (Ref: *no*)	29.11	45.48	34.67	48.00	25.33	43.64	27.33	44.72	
% Heard of, seen or participated in campaigns or activities about IPV prevention in community or workplace (Ref: *no*)	84.22	36.49	82.67	38.00	83.33	37.39	86.67	34.11	
% Tested for HIV (Ref: *have not been tested for HIV/unknown or missing/refused to answer*)	57.56	49.48	57.33	50.00	57.33	49.62	58.00	49.52	
% HIV Positive (Ref: *HIV Negative*; *HIV status unknown*; *refused to answer*)	3.11	17.38	4.67	21.00	2.00	14.05	2.67	16.16	
Attitudes on gender norms and relations (GEM scores)	44.20	6.54	43.31	6.80	44.74	6.23	44.55	6.53	
Household (HH) characteristics:									
Number of biological children	4.06	3.55	3.98	4.75	4.51	2.69	3.71	2.83	
Number of male children	1.94	1.49	1.67	1.51	2.34	1.41	1.81	1.47	***
Number of female children	1.71	1.44	1.52	1.34	1.99	1.57	1.61	1.37	**
% HH with food shortages of 3–12 months in a year (Ref: *never*)	44.44	49.75	45.33	49.95	45.33	49.95	42.67	49.62	
% HH members go without food because of lack of money (Ref: *never*)	24.22	42.89	22.00	41.56	26.00	44.01	24.67	43.25	
% Difficult to find money for treatment or medicine (Ref: *easy*)	70.22	45.78	74.67	43.64	67.33	47.06	68.67	46.54	
% Saves intermittently or never (Ref: *saves daily, weekly or monthly*)	49.11	50.05	50.00	50.17	48.67	50.15	48.67	50.15	
% Belongs to bottom 25th wealth quintile	24.67	43.16	23.33	42.44	31.33	46.54	19.33	39.62	*
% Belongs to 50th wealth quintile	23.78	42.62	12.67	33.37	35.33	47.96	23.33	42.44	***
% Belongs to 75th wealth quintile	25.11	43.41	20.00	40.13	25.33	43.64	30.00	45.98	
% Belongs to top 25th wealth quintile	26.44	44.15	44.00	49.80	8.00	27.22	27.33	44.72	***
Couples’ concordant reporting:									
Physical IPV	64.44	47.92	71.33	45.37	58.67	49.41	63.33	48.35	!
Sexual IPV	59.78	49.09	62.00	48.70	61.33	48.86	56.00	49.80	
Emotional IPV	52.00	48.85	45.33	49.95	60.00	49.15	50.67	50.16	*
Economic IPV	60.89	50.02	62.00	48.70	57.33	49.62	63.33	48.35	

***p ≤ 0.001; **p ≤ 0.01; *p ≤ 0.05; !*p* ≤ 0.10.

#### Physical IPV

In Intervention Arm 1, 78% of couples reported physical IPV occurrences concordantly at endline, a 31% increase from 60% at baseline (*p* = 0.002). In Intervention Arm 2, 24% of couples reported concordantly at endline, an increase of 24% from 62% at baseline (*p* = 0.02). In the Control Arm, 7% reported concordantly at endline, a 76% increase from 71% at baseline (*p* = 0.42).

#### Sexual IPV

Compared to baseline, 24% more couples in Intervention Arm 1 (*p* = 0.01), 22% more in Intervention Arm 2 (*p* = 0.04), and 10% more in Control Arm (*p* = 0.38) reported concordantly about sexual IPV at endline.

#### Economic IPV

Compared to baseline, 22% more couples in Intervention Arm 1 reported concordantly at endline (*p* = 0.05). Surprisingly, 10% fewer couples in Intervention Arm 2 (*p* = 0.32) and 13% fewer in Control Arm (*p* = 0.25) reported concordantly at endline.

#### Emotional IPV

From baseline to endline, concordant reporting of emotional IPV increased by 1% in Intervention Arm 1 (*p* = 0.90), 5% in Intervention Arm 2 (*p* = 0.68), and 30% in Control Arm (*p* = 0.09).

Table 3 presents the change in the rates of couples’ concordant reporting, *yes–no* discordance rate and *no–yes* discordance rate from baseline to endline, by study arm.

**Table 3:** Percent of couples reporting concordantly on IPV in the baseline survey and the endline survey, Tanzania, 2015–2016.

IPV	Control (*n* = 99)		*p > t*	Intervention 1 (*n* = 121)		*p > t*	Intervention 2 (*n* = 105)		*p > t*
	Baseline	Endline		Baseline	Endline		Baseline	Endline	
Physical IPV	70.71	75.76	0.42	59.50	77.69	0.00	62.00	77.14	0.02
Sexual IPV	61.62	67.68	0.38	61.98	76.86	0.01	60.00	73.33	0.04
Emotional IPV	40.40	52.53	0.09	60.33	61.16	0.90	57.14	60.00	0.68
Economic IPV	63.64	55.56	0.25	56.20	68.60	0.05	66.67	60.00	0.32

***p ≤ 0.001; **p ≤ 0.01; *p ≤ 0.05; †*p* ≤ 0.10.

#### Physical IPV

A higher reduction in yes–no discordance rate than in no–yes discordance rate was responsible for an increase in the rate of couples’ concordant reporting for three out of five acts of physical IPV in both Intervention Arm 1 (*slapped a partner or thrown something at her that could hurt her*; *pushed or shoved a partner*; *kicked, dragged, beaten, choked or burned a partner*), Intervention Arm 2 (*pushed or shoved a partner*; *hit a partner with a fist or with something else that could hurt her*; *kicked, dragged, beaten, choked or burned a partner*), and for one act in the Control Arm (*pushed or shoved a partner*), garnering greater concordance in couples’ reporting of overall physical IPV for Intervention Arms 1 and 2 compared to the Control Arm.

#### Sexual IPV

A higher reduction in *yes–no discordance* rate than in *no–yes discordance* rate was responsible for an improvement in the rate of couples’ concordant reporting for one (*forced a partner to have sexual intercourse when she did not want to*) out of two acts of sexual IPV across all arms.

#### Emotional IPV

A higher reduction in *yes–no* discordance rate than in *no–yes* discordance rate was responsible for an improvement in the rate of couples’ concordant reporting for four out of five acts of emotional IPV in Intervention Arm 1 (*insulted a partner or deliberately made her feel bad about herself*; *belittled or humiliated a partner in front of other people*; *done things to scare or intimidate a partner on purpose*; *hurt people she cares about as a way of hurting her*, or *damaged things that are important to her*), for three acts in Intervention Arm 2 (*belittled or humiliated a partner in front of other people*; *done things to scare or intimidate a partner on purpose*; *hurt people she cares about as a way of hurting her*, or *damaged things that are important to her*), and for one act in Control Arm (*done things to scare or intimidate a partner on purpose*).

#### Economic IPV

A higher reduction in *yes–no* discordance rate than in f: *no*, m: *yes* discordance rate was responsible for an improvement in couples’ concordant reporting for four out of four acts of economic IPV in Intervention Arm 1 (*prohibited a partner from getting a job, going to work, trading or earning money*; *taken a partner’s earnings against her will*; *thrown a partner out of her house*; *kept money from a partner’s earnings for alcohol, tobacco, or other things knowing that partner was finding it hard to afford household expenses*). Concordance grew worse (*prohibited a partner from getting a job, going to work, trading or earning money*; *taken a partner’s earnings against her will*; *thrown a partner out of her house*) or remained the same (*kept money from a partner’s earnings for alcohol, tobacco or other things knowing that partner was finding it hard to afford household expenses*) for all four acts of economic IPV in Intervention Arm 2, primarily due to an increase in *yes–no* discordance. The same pattern was also evident in the Control Arm.

### Regression findings

Table 4 presents results from two regression analyses: (1) comparing couples’ concordant reporting at the endline, by study arms, and (2) comparing changes from discordant to concordant reporting between baseline and endline by study arm. Compared to couples in the Control Arm, those in Intervention Arm 1 had higher odds of reporting concordantly on physical (OR: 1.32; *p* = 0.45), sexual (OR: 1.61; *p* = 0.095), emotional (OR: 1.02; *p* = 0.94), and economic IPV (OR: 1.76; *p* = 0.03), at the endline. In addition, compared to couples in the Control Arm, those in Intervention Arm 2 had higher odds of reporting concordantly on physical (OR: 1.18; *p* = 0.65), sexual (OR: 1.16; *p* = 0.58), and economic IPV (OR = 1.03; *p* = 0.92), and lower odds of reporting concordantly on emotional IPV (OR: 0.86; *p* = 0.54). Furthermore, compared to couples in the Control Arm, those in Intervention Arm 1 had a higher odds of changing from discordant to concordant reporting between the baseline and the endline on physical (OR: 1.85; *p* = 0.06), sexual (OR: 1.34; *p* = 0.38), and economic (OR: 1.83; *p* = 0.07) IPV, and had a lower odds of changing from discordant to concordant reporting between the baseline and the endline on emotional IPV (OR:0.58; *p* = 0.09). Interestingly, couples in Intervention Arm 2 were comparable to Control couples in terms of their odds of changing from discordant to concordant reporting between the baseline and the endline.

**Table 4:** Sources of increase in the rate of couples’ concordant reporting from baseline to endline by study arm, Tanzania, 2015–2016.

Physical IPV					Emotional IPV				
Study Arm	IPV act	Concordance	*Yes-no* discordance	*No-yes discordance*	Study Arm	IPV act	Concordance	*Yes-no* discordance	*No-yes discordance*
Control (*n* = 99)	Slapped/Thrown	−4%	6%	−2%	Control (*n* = 99)	Insulted	13%	−3%	−10%
	Pushed/Shoved	2%	1%	−3%		Public Insult	−3%	3%	NC*
	Hit with Fists	−1%	4%	−3%		Provoking Fear	2%	−6%	4%
	Kicked/Dragged	−4%	4%	NC*		Threatened	2%	1%	−3%
	Pointed Gun	−2%	2%	NC*		Displacement	−4%	2%	2%
	Physical IPV	4%	−2%	−2%		Emotional IPV	13%	−5%	−8%
Intervention 1 (*n* = 121)	Slapped/Thrown	12%	−7%	−5%	Intervention 1 (*n* = 121)	Insulted	4%	−4%	NC*
	Pushed/Shoved	15%	−11%	−4%		Public Insult	3%	−4%	1%
	Hit with Fists	NC*	NC*	NC*		Provoking Fear	10%	−11%	1%
	Kicked/Dragged	7%	−7%	−1%		Threatened	3%	−2%	−2%
	Pointed Gun	−5%	5%	NC*		Displacement	8%	−7%	−1%
	Physical IPV	18%	−14%	−4%		Emotional IPV	1%	NC*	−1%
Intervention 2 (*n* = 105)	Slapped/Thrown	10%	−4%	−6%	Intervention 2 (*n* = 105)	Insulted	6%	−2%	−4%
	Pushed/Shoved	18%	−12%	−6%		Public Insult	6%	−6%	NC*
	Hit with Fists	3%	−4%	1%		Provoking Fear	10%	−12%	3%
	Kicked/Dragged	1%	−3%	2%		Threatened	3%	NC*	−3%
	Pointed Gun	−4%	3%	1%		Displacement	1%	−4%	3%
	Physical IPV	16%	−9%	−8%		Emotional IPV	2%	−3%	1%

Physical IPV					Emotional IPV				
Study Arm	IPV act	Concordance	*Yes-no* discordance	*No-yes discordance*	Study Arm	IPV act	Concordance	*Yes-no* discordance	*No-yes discordance*
Control (*n* = 99)	Prohibits emp.	NC*	−5%	5%	Control (*n* = 99)	Forced Sex	9%	−11%	2%
	Takes money	−3%	8%	−5%		Humiliating Sex	3%	1%	−4%
	Thrown out	6%	−1%	−5%		Sexual IPV	5%	−6%	1%
	Controls spending	−6%	6%	NC*	Intervention 1 (*n* = 121)	Forced Sex	7%	−7%	1%
	Economic IPV	−8%	7%	1%		Humiliating Sex	1%	NC*	−1%
Intervention 1 (*n* = 121)	Prohibits emp.	10%	−6%	−4%		Sexual IPV	15%	−14%	−1%
	Takes money	−3%	−2%	5%	Intervention 2 (*n* = 105)	Forced Sex	11%	−13%	2%
	Throws out	12%	−9%	−3%		Humiliating Sex	−4%	4%	NC*
	Controls spending	6%	−3%	−2%		Sexual IPV	14%	−16%	2%
	Economic IPV	12%	−8%	−4%					
Intervention 2 (*n* = 105)	Prohibits emp.	−6%	6%	NC*					
	Takes money	−11%	3%	9%					
	Throws out	−7%	3%	4%					
	Controls spending	NC*	3%	−3%					
	Economic IPV	−7%	2%	5%					

*NC, No Change.

## Discussion and conclusion

To our knowledge, this is the first study to evaluate the feasibility of an intervention to improve agreement between couples’ reports of IPV incidents by influencing men’s reporting of their violent behavior. There is recognition of the need for data on IPV occurrences from both partners in a relationship for IPV prevention and mitigation, as acknowledged by the WHO in its “Ethical and safety recommendations for intervention research on violence against women” (25, 26). However, previous studies are mostly from high-income countries (27) and are observational, focusing on the level of disagreement or agreement in IPV reporting within couples or identifying risk factors (8, 15). In contrast, we tested an intervention intended to guide data collection strategies, with the goal of improving agreement.

We present evidence indicating that men’s violence reporting behavior can be influenced in settings where IPV is more socially accepted as a way to resolve conflicts within a couple’s relationship, and where IPV rarely results in legal consequences. While we observe some changes in men’s violence reporting behavior across all study groups—suggesting that some modifications may occur over time without any intervention—we find more significant and systematic changes, especially among men in Intervention Arm 1. It is important to note that most of these men adhered to intervention activities. On average, they attended 19 h of the male peer-group workshop, and 73% attended at least 18 h. Consequently, these men are more likely to participate fully in the intervention, increasing their awareness of their physical abuse and/or improving their ability to recognize behaviors, such as sexual and economic actions toward their female partners, as forms of abuse. Men tended to report violence more frequently and consistently to their female partners, especially when assured of confidentiality and anonymity.

Furthermore, we find that men’s reporting behavior is as modifiable for emotional violence as it is for physical, sexual, and economic violence through interventions. Despite this, our findings show that couples’ overall concordant reporting of emotional IPV increased only slightly, by 1% in Intervention Arm 1 and 1% in Intervention Arm 2 from baseline to endline, compared to a 30% increase in the Control Arm. In our item-by-item analysis of all five items in the emotional IPV module, we observed that the concordance rates—*no–yes* and *yes–no* discordance—were mostly similar across all arms for acts such as *belittling or humiliating a partner in front of others*, *intentionally scaring or intimidating a partner*, *threatening to harm a partner*, or *hurting people she cares about as a way of hurting her*, or *damaging things important to her*. However, for the item *that insulted a partner or deliberately made her feel bad about herself,* there was a 13-percentage-point increase in couples’ concordant reporting in the Control Arm from baseline to endline. This was driven by a larger decrease in the *no–yes* discordance rate compared to the *yes-no* discordance rate. Such a change in women’s reporting behavior is unlikely in Intervention Arms 1 and 2 because male partners were the intervention targets.

Our study has two primary limitations: First, with our sample size, which we adopted from an existing trial, we lacked the recommended 80% power to detect the intended rate of change in couples’ concordant IPV reporting. We had the power to detect a 50% change in the reduction in couples’ discordant reporting, assuming a 45% baseline prevalence rate and an *α* level of 0.05. This may explain why, despite evidence of association in descriptive analyses (Tables 3, 4), several estimates of effect in the regression analysis (Table 5) were not statistically significant at the conventional level (0.05). Second, for men, the survey attrition rate was 18.9% across all arms, 24.7% in Control, 13.3% in Intervention 1, and 18.7% in Intervention 2. For women, the survey attrition rate was 16.2% across all arms, 18% in Control, 10.7% in Intervention 1, and 20.0% in Intervention 2. Overall, the men who were lost were younger, more likely to use condoms, and had multiple sexual partners. The women who were lost were younger, less likely to have been married previously, to pay a dowry/to receive a bride price from their partners, or to have been paid a bride price by their partners, or to use alcohol, more likely to be involved in IPV activities, and had multiple sexual partners. Nonetheless, differential survey attrition across study arms did not compromise the randomization balance and was, therefore, unlikely to affect the study findings.

**Table 5:** Logistic regression estimates of average treatment effects on couples’ concordant reporting, *n* = 450 couples, Tanzania, 2015–2016.

IPV	Intervention 1 *vs*. Control			Intervention 2 *vs*. Control		
	OR	95% CI	*p > |z|*	OR	95% CI	*p > |z|*
Couples’ concordant IPV reporting (Ref: discordant reporting)						
Physical IPV	1.32	(0.64, 2.72)		1.18	(0.58, 2.40)	
Sexual IPV	1.61	(0.92, 2.82)	*	1.16	(0.68, 1.98)	
Emotional IPV	1.02	(0.61, 1.70)		0.86	(0.52, 1.41)	
Economic IPV	1.76	(1.05, 2.97)	**	1.03	(0.63, 1.70)	
Change from couples’ discordant to concordant reporting (Ref: none or change from concordant to discordant reporting)						
Physical IPV	1.85	(0.97, 3.51)	*	1.36	(0.70, 2.63)	
Sexual IPV	1.34	(0.70, 2.58)		1.40	(0.73, 2.69)	
Emotional IPV	0.58	(0.31, 1.09)	*	0.81	(0.45, 1.47)	
Economic IPV	1.83	(0.96, 3.46)	*	1.00	(0.50, 2.01)	

**p ≤ 0.05; *p ≤ 0.10. All models are adjusted for men’s school attendance, women’s school attendance, baseline concordance rates, and household asset index.

Despite limitations, our findings were instructive and promising, offering guidance to improve IPV data collection in Tanzania and across Sub-Saharan Africa. Our findings suggest that adding a preamble to IPV questionnaires that prompts enumerators to define IPV by its different forms would significantly improve the validity of men’s IPV reports. Such an amendment should help gather more accurate data from men who may not disclose IPV perpetration because they do not recognize their behaviors as abusive due to a lack of understanding of what constitutes abuse or having higher thresholds for what they consider to be an abusive interaction. Our study provides evidence that improving men’s knowledge of IPV can lead to higher levels of IPV disclosure among men and concordant reporting among couples.

## Data Availability

The raw data supporting the conclusions of this article will be made available by the authors, without undue reservation.
